# Genome-Wide Survey and Expression Profiling of bZIP Transcription Factors in *Juglans mandshurica* Reveal Candidate Genes Involved in Floral Development, Light Stress, and Drought/Salt Tolerance

**DOI:** 10.3390/ijms27135770

**Published:** 2026-06-26

**Authors:** Meng Dang, Huijuan Zhou, Rui Wang, Peng Zhao

**Affiliations:** 1 Eco-Environmental Conservation Research Centre of Xin’an River Basin, College of Life and Environmental Sciences, Huangshan University, Huangshan City 245021, China; dangmeng13@163.com; 2 Key Laboratory of Resource Biology and Biotechnology in Western China, Ministry of Education, College of Life Sciences, Northwest University, Xi’an 710069, China; shqyxlb2020@163.com; 3 Xi’an Botanical Garden of Shaanxi Province, Institute of Botany of Shaanxi Province, Shaanxi Academy of Science, Xi’an 710061, China; zhouhuijuan@xab.ac.cn

**Keywords:** bZIP transcription factors, genome-wide analysis, abiotic stress, ABA-responsive genes, *Juglans mandshurica*

## Abstract

Basic-region leucine zipper (bZIP) transcription factors are crucial for plant stress responses, but their characterization in the wild species *Juglans mandshurica* remains limited. Here, we identified 80 *bZIP* genes in the *J. mandshurica* genome and classified them into 13 subgroups, with notable enrichment in subgroups S, A, D, and I. All subgroup D members contain both bZIP and DELAY OF GERMINATION 1 (DOG1) domains, forming characteristic dual-module fusion proteins. Evolutionary analysis detected three orthologous gene pairs under positive selection since divergence from *Juglans regia*. Promoter cis-elements, especially MYB and MYC motifs, are abundant in *JmbZIP* genes. Protein–protein interaction networks suggest potential functional specialization and coordination among JmbZIP members. Expression profiling revealed distinct patterns across subgroups, with S, A, and D showing high activity across various physiological processes and light stress responses. qRT-PCR validated the dynamic expression of six ABA pathway marker genes, the ABRE-rich *JmbZIP41* and *JmbZIP42* genes, together with the highly expressed *JmbZIP12* gene under salt and drought stress. Our genome-wide analysis enabled the functional screening of bZIP members across subgroups. The key genes identified in this study provide valuable genetic resources for stress-resistance breeding in forest trees, with *JmABI5* (*JmbZIP40*) and *JmbZIP42* serving as prime candidates for enhancing tree stress tolerance.

## 1. Introduction

Transcription factors (TFs) are proteins that bind specifically to cis-acting elements in DNA to regulate the expression of target genes, playing pivotal roles in various biological processes in eukaryotes [1,2]. Among them, the basic region/leucine zipper (bZIP) family is named for its characteristic domain, which consists of a basic region rich in basic amino acids and a leucine zipper motif. The basic region, which contains an N-x7-R/K-x9 motif, is responsible for sequence-specific DNA binding and also functions as a nuclear localization signal [3]. The C-terminal leucine zipper region is composed of hydrophobic amino acid repeats, typically with a leucine residue at every seventh or ninth position, which form an amphipathic α-helix. This structure facilitates protein dimerization via van der Waals interactions. The combinatorial potential of dimerization, along with the presence of an acidic activation domain in some bZIPs, enables these TFs to recognize and bind cis-elements containing an ACGT core, such as ABRE, G-box, C-box, and A-box motifs [2]. Through such mechanisms, bZIP transcription factors critically influence plant development, stress responses, and the biosynthesis of secondary metabolites [4].

bZIP transcription factors function as crucial switches in eukaryotic transcriptional networks and represent one of the largest and most versatile transcription factor families in plants [5,6]. Genomic analyses reveal considerable variation in bZIP family size across plant species, with 78 in *Arabidopsis* [2], 99 in poplar [7], 85 in wheel wingnut [8], 227 in wheat [9], and 247 in rapeseed [10]. This expansion is primarily attributed to evolutionary mechanisms such as whole-genome duplication, segmental duplication, and tandem duplication. Systematic classification of the bZIP family has significantly advanced functional studies. The *Arabidopsis* bZIP transcription factor family has been genomically identified and systematically categorized into 13 subgroups (A-M) [2]. Research has indicated that members within the same subgroup often exhibit functional conservation and participate in regulating diverse physiological and metabolic processes. Among the numerous pathways modulated by bZIP proteins, their role in abscisic acid (ABA) signaling is particularly critical [11]. Studies in *Arabidopsis* have demonstrated that multiple bZIP subgroups, especially subgroup A (e.g., ABI5, ABF/AREBs), serve as core components of the ABA signaling pathway [12,13]. These transcription factors directly activate a suite of stress-responsive genes by binding to ABA-responsive elements, thereby playing a central regulatory role in plant adaptation to abiotic stresses such as drought and high salinity.

*Juglans mandshurica* (commonly known as Manchurian walnut) is a perennial deciduous tree of the Juglandaceae family, valued for its high-quality timber and remarkable adaptability to cold, drought, and salinity [14,15]. It also serves as an important rootstock resource for economic species such as *J. regia* and *Carya* spp. In *J. regia*, the bZIP gene family has been identified and characterized, with studies focusing on floral bud differentiation [16], explant browning [17], and the functional validation of *JrbZIP40* in salt and drought tolerance via transgenic *Arabidopsis* [18]. However, these investigations have been confined to *J. regia*, and the bZIP family in *J. mandshurica*, a wild relative with distinct genetic background and stress tolerance traits, remains entirely uncharacterized. Moreover, previous *Juglans* studies have largely centered on developmental processes or single stress-related genes, lacking a systematic examination of bZIP-mediated stress responses across multiple abiotic conditions.

Here, we present a genome-wide identification and systematic analysis of the bZIP transcription factor family in *J. mandshurica*. We comprehensively characterized its member composition, phylogenetic classification, gene structure, cis-elements, collinearity relationships, and protein–protein interaction networks. Furthermore, we investigated their expression patterns using transcriptome data from diverse tissues, light-stressed samples, and developing flowers. Key candidates were then validated by qRT-PCR under salt and drought stress. Overall, this study provides a foundational resource for the bZIP family in *J. mandshurica* and offers insights into its adaptive evolutionary patterns under natural selection pressure.

## 2. Results

### 2.1. Identification of bZIP Gene Family Members and Analysis of Protein Physicochemical Properties in Juglans mandshurica

**Figure 1:**
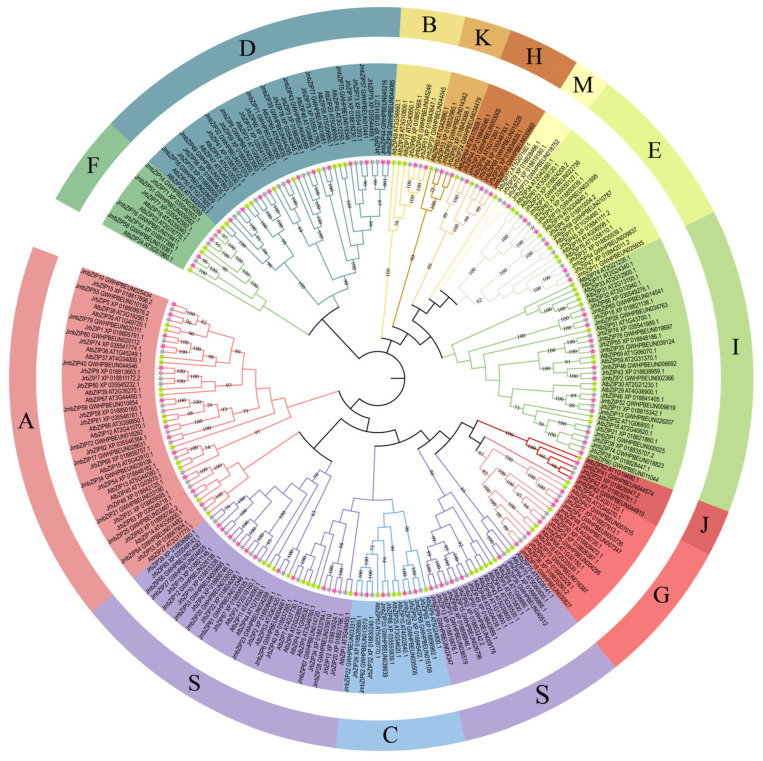
Maximum likelihood (ML) phylogenetic tree of bZIP proteins from *J. mandshurica*, *A. thaliana*, and *J. regia*. The tree was constructed using IQ-TREE v.2 based on MAFFT alignment of full-length protein sequences, with bootstrap support values (1000 replicates; ≥70% shown) at the branch nodes. Different colored background regions represent distinct subgroups, labeled A through S. JmbZIPs, AtbZIPs, and JrbZIPs are marked with magenta, green, and gray stars, respectively.

A total of 80 *bZIP* gene family members were identified in *J. mandshurica* and renamed according to their chromosomal locations. A maximum likelihood (ML) phylogenetic tree was constructed using bZIP family members from *Arabidopsis thaliana*, *Juglans regia*, and *J. mandshurica*, which classified all members into thirteen subgroups: A, B, C, D, E, F, G, H, I, J, K, M, and S (Figure 1). This classification is highly consistent with that of the bZIP family in *A. thaliana*. The distribution of *JmbZIP* members across subgroups was uneven (Figure S1). Subgroup S was the largest subgroup, comprising 15 members (18.75% of the total family). Subgroups A and D were the second-largest, each containing 12 members. Subgroup I included 11 members (13.75%). Together, these four subgroups (S, A, D, and I) accounted for 62.5% of all JmbZIP members.

### 2.2. Physicochemical Properties and Subcellular Localization Prediction of JmbZIP Proteins

The JmbZIP proteins showed considerable variation in their physicochemical properties. As shown in Table S1, protein lengths ranged from 145 amino acids (JmbZIP23) to 783 amino acids (JmbZIP37), with an average length of 339.19 amino acids. Predicted molecular weights ranged from 16.4 kDa (JmbZIP23) to 84.9 kDa (JmbZIP37), with an average of approximately 37.4 kDa. About half of the JmbZIP proteins had molecular weights between 20 and 45 kDa. Group-specific averages varied from 20.0 kDa (subgroup S) to 83.7 kDa (subgroup B). Isoelectric point analysis revealed that the majority of JmbZIP proteins had pI values between 4.80 and 10.04. Instability index predictions indicated that the majority of members had instability indices exceeding 40. The average hydrophilicity index of all JmbZIP proteins was negative. Subcellular localization analyses predicted that all JmbZIP proteins are localized in the nucleus (Table S1).

### 2.3. Chromosomal Distribution and Duplication Patterns of the JmbZIP Gene Family

**Figure 2:**
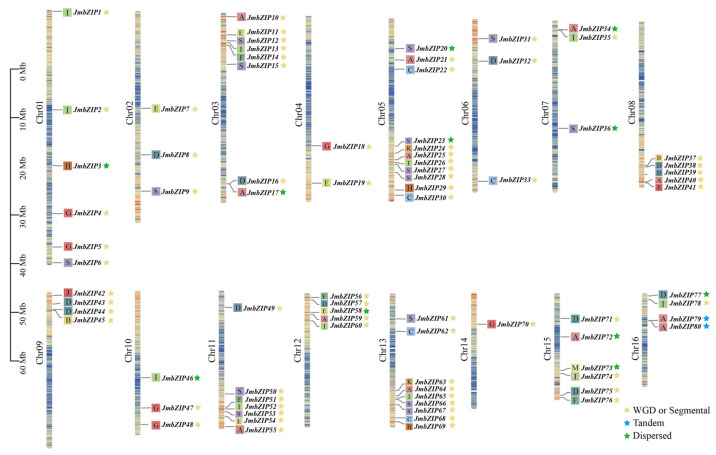
Chromosome distribution of the bZIP gene family in *J. mandshurica*. Chromosomes are numbered 1–16 according to the assembly accessions GWHBEUN00000001-16.

The *JmbZIP* genes exhibited a non-uniform distribution across the chromosomes of *J. mandshurica* (Figure 2; Table S1). Chr5, Chr13, and Chr3 contained the highest numbers of genes, with 11 (13.75%), 9 (11.25%), and 8 (10.0%) genes, respectively. Together, these three chromosomes accounted for more than one-third of all *JmbZIP* genes. In contrast, Chr14 contained only one gene (1.25%), making it the most gene-sparse chromosome. Different subgroups exhibited distinct chromosomal distribution preferences (Figure S2). Chr5 contained the highest number of genes and the most diverse set of subgroups (Figure S2).

To investigate the evolutionary expansion mechanisms of the *JmbZIP* gene family in *J. mandshurica*, we analyzed its duplication modes using the Multiple Collinearity Scan toolkit (MCScanX v1.0.0). Among the 80 *JmbZIP* genes, two (*JmbZIP79* and *JmbZIP80*) were classified as tandem duplicates, and 11 (*JmbZIP3*, *JmbZIP17*, *JmbZIP20*, *JmbZIP23*, *JmbZIP34*, *JmbZIP36*, *JmbZIP46*, *JmbZIP58*, *JmbZIP72*, *JmbZIP73*, and *JmbZIP77*) were classified as dispersed duplicates. The remaining genes were identified as originating from whole-genome duplication or segmental duplication events (Figure 2; Table S2). These results suggest that whole-genome or segmental duplication may have been the primary driver of *JmbZIP* family expansion in *J. mandshurica*.

### 2.4. Conserved Domains and Gene Structure of JmbZIP Members

**Figure 3:**
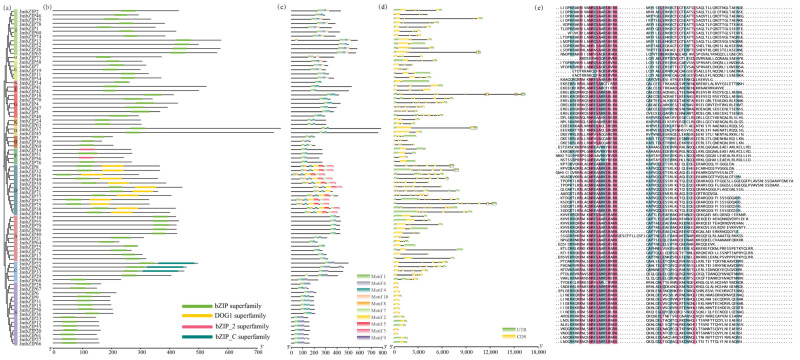
Conserved domains and gene structure of JmbZIP members: (**a**) Maximum likelihood (ML) phylogenetic tree of JmbZIPs. (**b**) Protein domains of JmbZIPs, with different domains indicated by different colored boxes. (**c**) Motif analysis of JmbZIPs, with different motifs indicated by different colored boxes. (**d**) Gene structure of *JmbZIPs*, where green boxes represent UTRs, yellow boxes represent CDS, and gray lines represent introns. (**e**) Amino acid sequence alignment of the bZIP domains. Residue shading reflects conservation levels (pink: ≥75%; light blue: ≥50% homology).

The phylogenetic tree constructed from full-length protein sequences using the maximum likelihood method showed a topology similar to that of the cross-species tree containing bZIP members from *A. thaliana* and *J. regia* (Figure 3a). Domain annotation revealed that most JmbZIP proteins harbor a conserved bZIP domain (bZIP superfamily). All members of subgroup D possess both the bZIP and DELAY OF GERMINATION 1 (DOG1) domains, forming ‘dual-module’ fusion proteins (Figure 3b). Conserved motif analysis identified 10 motifs. Motifs 1, 4, and 6 are located within the bZIP domain, whereas motifs 2, 3, 5, 7, and 8 are located within the DOG1 region (Figure 3c). The motif composition was largely consistent within each subgroup, which is consistent with the phylogenetic grouping. Gene structure analysis revealed considerable variation in intron number among *JmbZIP* genes, but conserved patterns within subgroups, corresponding to the phylogenetic classification (Figure 3d). The bZIP domains of all 80 JmbZIP members were extracted and aligned to investigate sequence conservation. As shown in Figure 3e, all proteins contain the canonical bZIP domain comprising a basic region and a leucine zipper. The basic region is highly conserved across all groups. The leucine zipper region exhibits the characteristic heptad repeat pattern, with leucine residues occurring at every seventh position. The group assignments based on the full-length phylogenetic tree correspond to the conservation patterns in the bZIP domain.

### 2.5. Selective Pressure Analysis of bZIP Orthologs and Paralogs in Juglans

**Figure 4:**
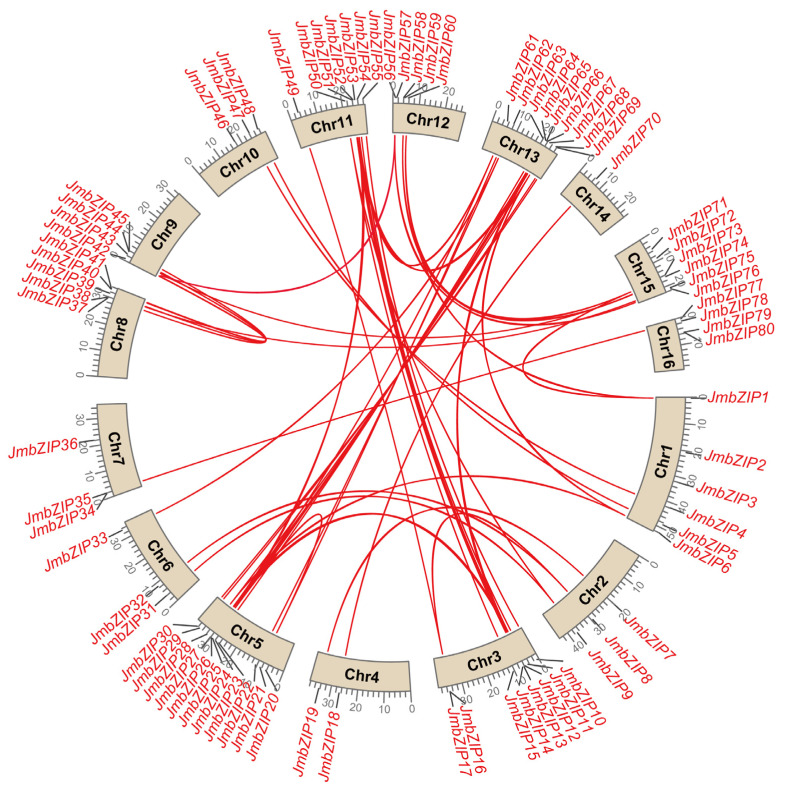
Collinearity analysis of the bZIP gene family in *J. mandshurica*. Red lines connect bZIP gene pairs that share syntenic relationships.

To investigate the selective pressures on the bZIP gene family, we first identified syntenic bZIP gene pairs within the *J. mandshurica* genome and between *J. mandshurica* and *J. regia* through collinearity analysis (Figure 4 and Figure S3). Based on these syntenic pairs, we performed Ka/Ks analysis on 159 orthologous gene pairs between the two species and 50 paralogous pairs within the *J. mandshurica* genome (Tables S3 and S4). The analysis revealed that the vast majority of both orthologous and paralogous pairs had Ka/Ks values significantly less than 1, suggesting that the bZIP family has been predominantly subject to purifying selection. In contrast, three orthologous pairs (*JmbZIP24* vs. *JrbZIP50*, *JmbZIP64* vs. *JrbZIP53*, and *JmbZIP66* vs. *JrbZIP85*) exhibited Ka/Ks values greater than 1, suggesting positive selection on these gene pairs.

### 2.6. Analysis of Cis-Regulatory Elements in JmbZIP Promoters

**Figure 5:**
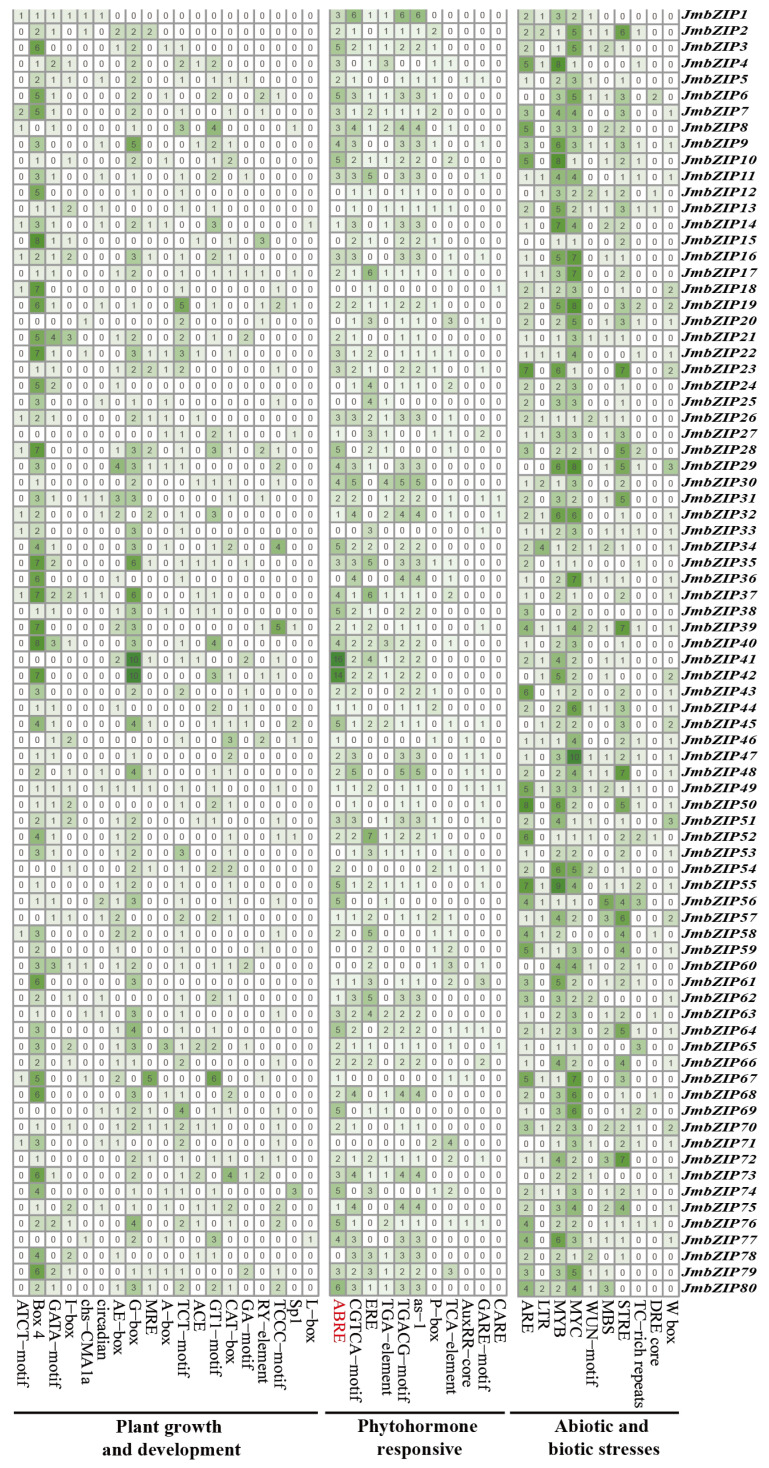
Cis-acting element analysis in *JmbZIP* promoters. ABRE is highlighted in red as the cis-acting element of particular interest in this study.

To investigate the cis-regulatory elements in the promoter regions of *JmbZIP* genes, we analyzed the 2 kb upstream sequences of all 80 members. These elements were classified into three major categories: plant growth and development, phytohormone response, and abiotic/biotic stress response (Figure 5). The promoters of *JmbZIP* genes contained stress-responsive elements such as ARE, MYB, MYC, and STRE. *JmbZIP41* and *JmbZIP42* (subgroup J) harbored 16 and 14 copies of ABA-responsive elements (ABREs), respectively. Both genes are orthologous to *AtbZIP62* (*AT1G19490.1*) and were selected for further expression analysis under salt and drought stress responses.

### 2.7. Expression Analysis of JmbZIP Genes

**Figure 6:**
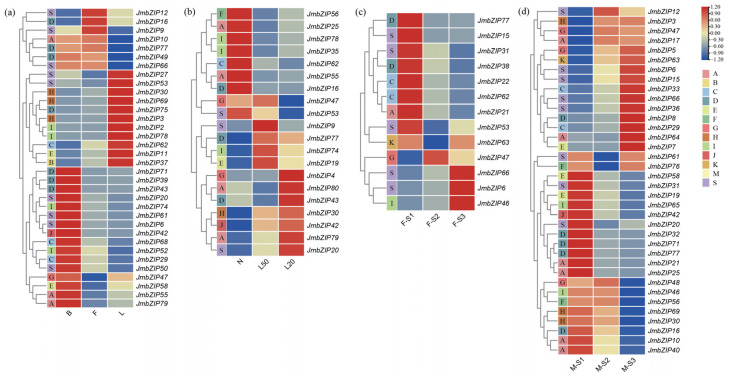
Expression patterns of the *JmbZIP* gene family across various conditions: (**a**) Baseline expression in bark (B), green pericarp (F), and leaf (L) tissues. (**b**) Response to a 6 h exposure to different light intensities (100%, 50%, and 20% of full sunlight). (**c**,**d**) Dynamic expression during the three developmental stages of female (F-S1 to F-S3) and male (M-S1 to M-S3) flowers. The heatmap shows Z-score-normalized expression levels of the differentially expressed *JmbZIP* genes (|log_2_FC| ≥ 1, FDR ≤ 0.05). Subgroup affiliations are indicated by the color bar on the right.

To investigate the expression patterns of *JmbZIP* genes across different tissues and under light stress conditions, we analyzed publicly available RNA-seq datasets of *J. mandshurica* (Table S5). We first analyzed the baseline expression profiles of the *JmbZIP* family in bark (B), green fruit pericarp (F), and green leaves (L), revealing differential expression of 33 genes across these tissues (Figure 6a; Table S6). Subgroup S contained the most differentially expressed genes (DEGs), with 9 members, followed by subgroup D with 6. *JmbZIP12* (subgroup S) showed high expression levels across all three tissues, with elevated expression in the green fruit pericarp (FPKM = 450.70). *JmbZIP55* (subgroup A) showed tissue-specific expression, with high expression in bark (FPKM = 189.97) and low expression in the green pericarp (FPKM = 2.03).

Analysis of the *JmbZIP* family under light stress identified a series of responsive genes (Figure 6b; Table S7). *JmbZIP12*, *JmbZIP23*, and *JmbZIP27* (subgroup S) maintained high expression levels under all light conditions. *JmbZIP79* (subgroup A), *JmbZIP30* (subgroup H), and *JmbZIP4* (subgroup G) showed increased expression as light intensity decreased.

The distribution of differentially expressed genes (DEGs) among subgroups was consistent across the three developmental stages within each flower type (female and male; Figure 6c,d; Tables S8 and S9). Subgroup S contributed the most DEGs in both male (8 genes) and female (5 genes) flowers. Subgroups A and D also showed expression changes in both flower types. *JmbZIP66* (subgroup S) showed a similar expression pattern in both female and male flowers: low at early stages (F-S1/M-S1), increasing throughout development, and peaking at late stages (F-S3/M-S3; FPKM = 46.85 and 189.02 in female and male flowers, respectively). *JmbZIP12* (subgroup S) was expressed at high levels across all stages of both female and male flower development, maintaining peak levels in mid-to-late phases. *JmbZIP71* (subgroup D) initially showed high expression, which then declined in male flowers, while *JmbZIP20* (subgroup S) expression gradually decreased in female flowers.

### 2.8. Protein–Protein Interaction Analysis of JmbZIP Proteins

**Figure 7:**
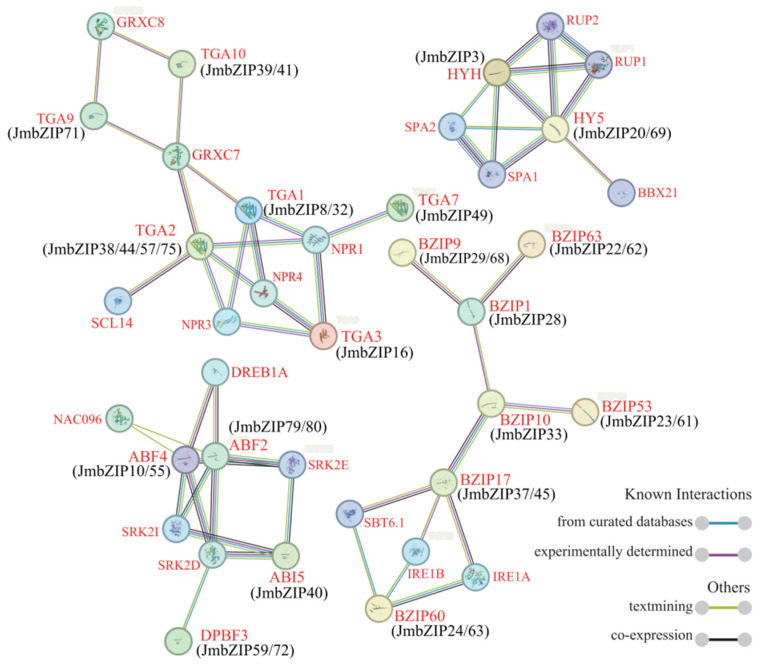
Predicted protein–protein interaction network of JmbZIP proteins. The network nodes represent proteins. The 3D structure of the protein is shown inside the nodes, and the line color indicates the type of interaction evidence.

To investigate the potential functions of JmbZIP proteins, we constructed a protein–protein interaction (PPI) network using the STRING database based on ortholog-based queries (Figure 7). The network revealed that subgroup D members (orthologous to *A. thaliana* TGA1/2/3/7/9/10 transcription factors) showed interactions with *NPR1* and its paralogues *NPR3/4*. This module also included connections to glutaredoxins GRXC7 and GRXC8. Subgroup A members (orthologous to *A. thaliana* ABF transcription factors, e.g., *ABF2/4)* showed interactions with proteins involved in ABA signaling and stress responses. Subgroup H members (orthologous to *Arabidopsis HY5*) showed interactions with light-signaling components (e.g., *BBX21*) and subunits of the COP1-SPA E3 ubiquitin ligase complex (e.g., *SPA1/2*). Subgroups S, C, and B were associated with *IRE1A*, *IRE1B*, and *SBT6.1*, which are involved in ER stress and protein processing.

### 2.9. Role of JmbZIP Genes in ABA-Mediated Salt and Drought Stress Responses

To analyze their expression under stress, this study identified key components of the ABA signaling pathway in *J. mandshurica*, including *JmNCED3*, *JmABI5* (*JmbZIP40*), *JmABF4* (*JmbZIP55*), *JmRAB18*, and *JmP5CS1*, along with *Jm4CL2*, a gene associated with stress-responsive phenylpropanoid metabolism. In addition, three *JmbZIP* genes (*JmbZIP12*, *JmbZIP41*, and *JmbZIP42*) were selected for qRT-PCR validation based on multi-dimensional criteria (detailed in the Discussion). Primers for the expression analysis of these genes are provided in Table S10. Conserved domain analysis confirmed that all selected genes encode predicted functional proteins. We analyzed the expression dynamics of these genes by qRT-PCR in leaves subjected to salt and drought treatments (Table S11).

**Figure 8:**
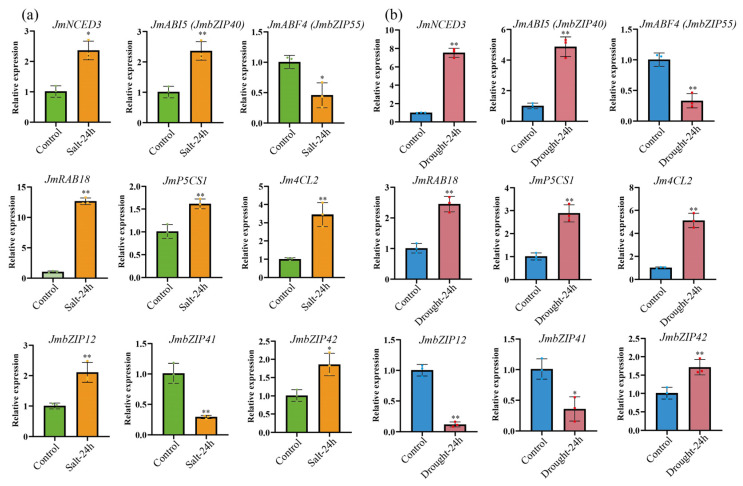
The expression patterns of selected genes verified by qRT-PCR. (**a**) Expression levels under salt stress. (**b**) Expression levels under drought stress. * (*p* < 0.05) and ** (*p* < 0.01) display the levels of significant differences. The error bars represent the SE of three independent experiments. Values are means ± SE.

**Figure 9:**
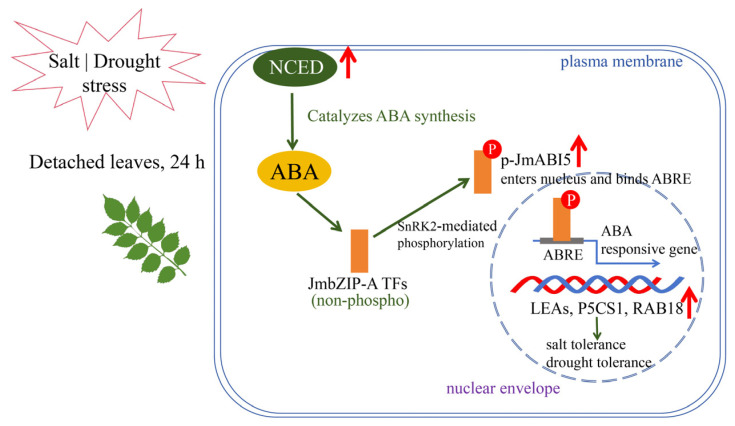
ABA-centered working model in *J. mandshurica* leaves. Salt/drought induces NCED to boost ABA biosynthesis, thereby activating ABRE-dependent stress genes. Upward arrows indicate increased abundance or activity.

The expression of key ABA pathway genes, with the exception of *JmABF4*, was upregulated 24 h after both salt and drought treatments (Figure 8). *JmNCED3* expression was induced approximately 2.7-fold under salt stress and 7.5-fold under drought stress (Figure 8 and Figure 9). *JmABI5* transcript levels were induced, which correlated with the upregulation of *JmRAB18* and *JmP5CS1*. In contrast, *JmABF4* expression was downregulated. *Jm4CL2* was induced approximately 3.4-fold and 5.1-fold under salt and drought stress, respectively (Figure 8). *JmbZIP42* and *JmbZIP41* (subgroup J) showed contrasting expression patterns, while *JmbZIP12* showed distinct responses to salt versus drought stress (Figure 8).

## 3. Discussion

A total of 80 bZIP transcription factors were identified in *Juglans mandshurica* and phylogenetically classified into 13 subgroups. Notably, subgroups S, A, D, and I collectively contained over half of all members, suggesting their crucial roles in stress response and developmental regulation. All subgroups contained members from JmbZIPs, AtbZIPs, and JrbZIPs, with genes within the same subgroup clustering together [2,16]. This strongly implies that these genes originated from common ancestors and their basic functions were established prior to the divergence of monocot and dicot plants and have been conserved throughout evolution. Subgroup S, the largest with 15 members, is involved in plant development, light signaling, and stress responses [2]. Its expansion suggests that *J. mandshurica* may possess a complex signaling network essential for its perennial woody life cycle. Subgroup A, with 12 members, serves as a core component of the ABA signaling pathway [19,20]. Its abundance indicates a robust ABA-dependent stress resistance foundation, supporting adaptation to variable environments. Subgroup D, also with 12 members, integrates multiple stress signals via the mitogen-activated protein kinase (MAPK) cascade and participates in endoplasmic reticulum stress and programmed cell death [21,22]. Its expansion may enhance the tree’s ability to cope with combined environmental stresses. Notably, this subgroup uniquely features a conserved bZIP-DOG1 dual-domain fusion, suggesting a specialized mechanism that involves coupling signal perception with DNA binding for precise transcriptional regulation [23]. Subgroup D also possesses its own unique conserved small motifs, implying regulatory mechanisms distinct from other bZIP subgroups. Additionally, subgroup G (6 members) and subgroup H (3 members) are key regulators of photomorphogenesis [24,25]. They play vital roles in seedling establishment, shade adaptation, and photosynthesis, providing candidate genes for elucidating light response mechanisms in *J. mandshurica*.

The clustered, non-random distribution of *JmbZIP* genes on chromosomes is likely associated with their functional differentiation, gene duplication events, and local chromatin environment, providing clues for deciphering the regulatory roles of key chromatin regions in stress responses. Analysis of duplication modes revealed that family expansion was primarily driven by whole-genome or segmental duplication events. This finding is consistent with the evolutionary patterns observed in many other plant gene families, reinforcing the crucial role of whole-genome duplication as a core engine for plant genome evolution and functional diversification of gene families [4,10,26]. The large-scale expansion and retention of the *J. mandshurica* bZIP family through ancient WGD events have established a structural foundation for complex transcriptional regulatory functions, which may have significantly enhanced environmental adaptability [27,28].

Cis-element profiling revealed that numerous *JmbZIP* promoters are enriched in stress-related motifs, particularly the MYB and MYC binding sites that function as core components of abscisic acid (ABA) signaling. As ABA is a master hormone in plant responses to drought, salinity, and low temperature, the prevalence of these motifs strongly implies that *JmbZIP* genes integrate ABA signals to orchestrate downstream stress-responsive pathways [20]. Most strikingly, subgroup J members *JmbZIP41* and *JmbZIP42* contain exceptionally high numbers of ABA-responsive elements (ABREs), the key cis-acting sites of ABA signaling (Figure 5). This striking ABRE enrichment positions these genes as prominent candidates for ABA-mediated stress responses [2]. Protein–protein interaction (PPI) network analysis revealed distinct functional specialization and synergy among family members, forming multiple functionally specific interaction modules (Figure 6). Collectively, the PPI network delineates a functional hierarchy in which subgroup D governs immune responses, while subgroups A, H, and S act synergistically against abiotic stresses [29,30].

Beyond the PPI-based predictions, multi-dimensional expression profiling revealed distinct roles of the JmbZIP family in growth, development, and stress adaptation (Figure 7 and Figure S4). Members of subgroups S, A, and D were consistently active across conditions, implying central regulatory functions. Under light stress, *JmbZIP30* (subgroup H; ortholog of *Arabidopsis HY5*) exhibited a dose-dependent increase in expression as light intensity decreased, suggesting that it transduces low-light signals to activate antioxidant defense genes, thereby mitigating photo-oxidative damage and maintaining redox homeostasis [25]. *JmbZIP55* (subgroup A), an ortholog of the core ABA signaling transcription factor *Arabidopsis ABF4*/*AREB2*, was predominantly expressed in bark. This specific expression pattern supports its proposed role as a regulatory hub that integrates ABA signals to confer protection in this exposed tissue [20]. *JmbZIP12*, *JmbZIP20*, and *JmbZIP66* (subgroup S; *Arabidopsis GBF5-like*) were detected in both organ development and stress responses. The constitutive high level of *JmbZIP12* suggests a housekeeping role in primary metabolism, whereas the sharp upregulation of *JmbZIP66* at late floral stages indicates a temporal function in reproductive development, illustrating divergence among GBF5 paralogues [31]. We also identified several genes with low basal expression but high fold changes, a hallmark of potent regulatory nodes that can orchestrate downstream responses through signaling cascades [32]. These candidates provide crucial entry points for elucidating the molecular mechanisms of agronomic traits and for future targeted breeding.

To prioritize candidates for experimental validation, we integrated phylogenetic relationships, cis-element profiles, and expression patterns. *JmbZIP12* (subgroup S) is closely related to *AtbZIP1*, a positive regulator of salt, osmotic, and drought tolerance [33]. *JmbZIP41* and *JmbZIP42* (subgroup J) are closely related to *AtbZIP62*, which positively regulates drought and ABA signaling but negatively regulates salt tolerance via the SOS pathway [34,35,36]. These evolutionary links, together with their exceptional ABRE enrichment (Figure 5) and stress-inducible expression patterns, provided a multi-dimensional rationale for selecting these three genes for qRT-PCR validation. In parallel, we examined the expression of known ABA signaling components. *JmABI5* (subgroup A) was strongly induced under salt and drought stress, with its upregulation correlating with increased transcript levels of downstream genes *JmRAB18* and *JmP5CS1*, supporting its role as a positive regulator in ABA-mediated stress responses [37]. In contrast, *JmABF4* expression was downregulated, suggesting a potential feedback-regulatory mechanism or predominant regulation at the protein level [37]. This selection is further supported by functional studies of homologous bZIP genes in other woody species. In *Cyclocarya paliurus* (Juglandaceae), *CpbZIP19* and *CpbZIP41* (orthologous to *AtABI5*) are upregulated under drought stress [8]. In grapevine, group A members *VvbZIP19/25/53* cluster with *AtbZIP39/ABI5* and are implicated in ABA-mediated processes [38]. In Chinese jujube, *ZjbZIP10* (subgroup A, clustered with *AtABI5*) shows increased expression under salt stress [39]. In poplar, *PtrbZIP3* (subgroup A) functions as an ABA-dependent transcriptional activator that binds ABRE and improves osmotic tolerance [40]. Notably, *JrbZIP40* (subgroup S) has been functionally validated to enhance salt and drought tolerance in transgenic *Arabidopsis* [18], supporting the selection of *JmbZIP12* (also subgroup S) as a candidate for stress response studies. Collectively, these findings across diverse woody taxa support the conserved roles of subgroup A and S bZIPs in ABA-mediated stress responses. Notably, *JmbZIP41* and *JmbZIP42*, both from subgroup J, exhibited opposing expression responses to stress, suggesting possible divergence within this subgroup. The key *JmbZIP* genes identified in this study provide valuable genetic resources for stress resistance breeding in forest trees, with *JmABI5* and *JmbZIP42* representing promising candidates for enhancing stress tolerance.

Based on homology to *Arabidopsis* and supported by our expression data, we propose a model for ABA-mediated stress responses in *J. mandshurica* (Figure 9). Under salt or drought stress, the significant induction of *JmNCED3* is predicted to elevate endogenous ABA levels, initiating the canonical ABA signaling cascade [41]. The significant upregulation of *JmABI5* and its positive correlation with the expression of downstream genes *JmRAB18* and *JmP5CS1* suggest that *JmABI5* likely functions as a positive regulator. Although direct DNA binding was not tested here, studies in *Arabidopsis* have demonstrated that *ABI5* homodimers directly bind the ABRE motif in the promoters of *RAB18* and *P5CS1* [42,43], supporting the hypothesis that *JmABI5* activates transcription in a similar manner. In contrast, the significant downregulation of *JmABF4* may indicate that its regulation occurs primarily at the post-transcriptional level. We speculate that the *JmABF4* protein might be activated early during stress, while the observed mRNA downregulation represents a feedback mechanism to prevent signaling overload [44,45]. In summary, this model places our qRT-PCR-derived expression patterns into a literature-supported mechanistic framework without claiming new protein–DNA interactions. Future work should focus on the functional validation of these predicted regulatory relationships, including DNA occupancy and kinase-mediated phosphorylation.

## 4. Materials and Methods

### 4.1. Identification of the *JmbZIP* Gene Family in *Juglans mandshurica*

Amino acid sequences of *Arabidopsis thaliana* bZIP proteins were downloaded from the TAIR database (https://www.arabidopsis.org/). Domain prediction for these *Arabidopsis* bZIP sequences was performed using NCBI CDD (https://www.ncbi.nlm.nih.gov/cdd/ (accessed on 13 August 2025)) and the Pfam database within InterPro (https://www.ebi.ac.uk/interpro/search/sequence/ (accessed on 13 August 2025)). Sequences containing both the PF00170 and PF07716 domains were retained. Subsequently, motif analysis was conducted using MEME (https://meme-suite.org/meme/tools/meme (accessed on 13 August 2025)) [46]. The final curated set of *Arabidopsis* bZIP sequences served as queries for a local BLASTP search (NCBI BLAST+ v.2.16.0) against the published chromosome-level genome of *J*. *mandshurica* [47] using TBtools v.2.467 software [48], yielding preliminary candidate *JmbZIP* genes. These candidate sequences were then submitted to CDD, Pfam, and SMART (http://smart.embl-heidelberg.de/ (accessed on 13 August 2025)) online databases to verify the integrity of the bZIP domain. Redundant, duplicated, and incompletely annotated sequences were removed. The final retained sequences underwent further motif analysis via MEME, ultimately identifying 80 candidate members of the *JmbZIP* gene family. Gene structure analysis and visualization for these *JmbZIP* family members were performed using TBtools v.2.467 software [48] and the *J. mandshurica* genome annotation file. The conserved domain sequences were extracted and subjected to multiple sequence alignment using DNAMAN v.9.0 (Lynnon Biosoft, Quebec City, QC, Canada). The alignment was visualized with shading to indicate residue conservation. The basic region and leucine zipper were delimited based on the canonical bZIP structure.

### 4.2. Prediction of Physicochemical Properties and Subcellular Localization of JmbZIP Proteins

The physicochemical properties of the JmbZIP proteins, including the number of amino acids, molecular weight, theoretical isoelectric point (pI), and grand average of hydropathicity (GRAVY), were predicted using the ExPASy ProtParam online tool (https://web.expasy.org/protparam/ (accessed on 14 August 2025)). Subcellular localization was predicted using the plant-mPLOC (http://www.csbio.sjtu.edu.cn/bioinf/plant-multi/ (accessed on 14 August 2025)) and TargetP-2.0 (https://services.healthtech.dtu.dk/services/TargetP-2.0/ (accessed on 14 August 2025)) online tools.

### 4.3. Phylogenetic Analysis of the JmbZIP Gene Family

The full-length protein sequences of bZIP members from *J. mandshurica*, *A. thaliana*, and *J. regia* were aligned using MAFFT v.7 with the --auto strategy [49]. The full MAFFT alignment was employed for phylogenetic reconstruction to preserve the most informative sites. Maximum likelihood (ML) trees were constructed using IQ-TREE v.2 with 1000 bootstrap replicates and the JTT+F+I+R6 model selected as the best-fit model according to the BIC [50]. Two independent ML trees were generated: one containing bZIP members from *J. mandshurica*, *A. thaliana*, and *J. regia* for cross-species classification and the other containing only the 80 JmbZIP members of *J. mandshurica* for intra-species analysis. Bootstrap values of ≥70% were considered strong support. The resulting trees were visualized and annotated using the online tool iTOL v.5 [51].

### 4.4. Chromosomal Distribution, Gene Duplication, and Synteny Analysis

*JmbZIP* genes were mapped onto chromosomes based on their physical positions in the *J. mandshurica* genome. The chromosomal location diagram was generated using the MG2C online software v2.0 (http://mg2c.iask.in/mg2c_v2.0/ (accessed on 17 August 2025)). Gene duplication events were analyzed using the MCScanX v1.0.0 with default parameters [52]. Synteny analysis was performed among the genomes of *J. mandshurica*, *A. thaliana*, and *J. regia*, with the results visualized using TBtools v.2.467 [48]. Furthermore, the KAKS_CALCULATOR v.2.0 software was used to calculate the non-synonymous (Ka) and synonymous (Ks) substitution rates for identified duplicated gene pairs [53].

### 4.5. Prediction and Analysis of Cis-Acting Elements

Promoter sequences, defined as the 2000 bp genomic regions upstream of the transcription start sites of the identified *JmbZIP* genes, were submitted to the PlantCARE database (http://bioinformatics.psb.ugent.be/webtools/plantcare/html/ (accessed on 25 August 2025)) for cis-acting element prediction. The results were statistically screened and visualized using a custom R script (R v.4.3.2).

### 4.6. RNA-Seq Data Processing and Differential Expression Analysis

This study utilized publicly available RNA-seq data from the NCBI SRA database (https://www.ncbi.nlm.nih.gov/) to investigate the transcriptional responses of the *JmbZIP* genes across different key tissues (bark, green pericarp, leaf) [47], light stress (100%, 50%, 20% full sunlight) [54], and during three developmental stages of female and male flowers [55] (Table S5). The downloaded SRA files were converted to FASTQ format. Raw reads were then quality-trimmed using Trimmomatic with parameters “LEADING:3 TRAILING:3 SLIDINGWINDOW:4:15 MINLEN:36”. The cleaned reads were aligned to the *J. mandshurica* reference genome using HISAT2 v.2.2.1 to generate bam files. Transcript abundance was quantified at the gene level using featureCounts v2.1.1 via an R script (run-featurecounts.R) to obtain a count matrix [56]. Differentially expressed genes (DEGs) were identified using a Perl script (run_DE_analysis.pl) implementing the DESeq2 v1.42.1 method with a significance threshold of FDR < 0.05 and |log2FoldChange| > 1. Heatmaps of DEGs were generated using TBtools v.2.467 [48].

### 4.7. Protein–Protein Interaction Network Analysis of JmbZIP Proteins

To construct a protein–protein interaction (PPI) network for JmbZIP proteins, the OrthoVenn2 online tool (https://orthovenn2.bioinfotoolkits.net/ (accessed on 20 August 2025)) was first used to identify the most homologous *A. thaliana bZIP* gene for each *JmbZIP* gene. Subsequently, using these *Arabidopsis bZIP* genes as seed sequences, their orthologs in *J. mandshurica* were identified with specific parameters (E-value: 1 × 10^−2^, Inflation value: 1.5). Finally, the PPI network for the JmbZIP proteins was predicted and visualized using the STRING database (https://string-db.org/).

### 4.8. Validation by Quantitative Real-Time PCR (qRT-PCR)

To investigate the potential roles of JmbZIP transcription factors in the ABA signaling pathway, qRT-PCR analysis was performed using *J. mandshurica* leaf samples subjected to salt and drought stress. Intact middle leaves from the 3rd–4th compound leaves of uniformly grown *J. mandshurica* seedlings were used for treatments. All leaves were selected with strict consistency in leaf position, size, developmental stage, and physiological status to ensure sample uniformity. For stress treatments, whole leaves were fully immersed in salt stress solution (200 mM NaCl) or drought-mimetic stress solution (20% *w*/*v* PEG-6000). The treatments were carried out in Petri dishes with gentle shaking (80 rpm) at 25 °C under a 16 h light/8 h dark photoperiod for 24 h. Control leaves were immersed in distilled water under identical conditions. After 24 h, all samples (including controls) were collected, immediately frozen in liquid nitrogen, and stored at −80 °C for total RNA extraction. Leaves were collected in late August 2025 from the resource garden of Xi’an Botanical Garden of Shaanxi Province (109.03° E, 34.21° N, Xi’an, China). RNA integrity, purity, and concentration were assessed by agarose gel electrophoresis and a NanoDrop™ spectrophotometer. After cDNA synthesis, six key ABA signaling pathway genes (*JmNCED3*, *JmABI5*, *JmABF4*, *JmRD29B*, *JmP5CS1*, and *Jm4CL2*) and three *JmbZIP* genes (*JmbZIP12*, *JmbZIP41*, and *JmbZIP42*) were selected for expression analysis (primer sequences listed in Table S10). The β-actin gene was used as an internal reference. Specific primers were designed using the Primer3Plus online website (https://www.primer3plus.com (accessed on 11 November 2025)) and synthesized by Sangon Biotech (Shanghai, China). The qRT-PCR reactions were carried out on a Bio-Rad CFX96 Real-Time PCR System (Bio-Rad, Hercules, CA, USA), with three technical replicates for each reaction. qRT-PCR results were analyzed by the 2^−ΔΔCT^ method [57]. All data are presented as means ± standard error (SE). Statistical significance between the treated and control groups was assessed using Student’s *t*-test. Prior to analysis, data normality and homogeneity of variances were confirmed using the Shapiro–Wilk and Levene’s tests, respectively. A value of *p* < 0.05 was considered statistically significant. Three independent biological replicates were performed for each treatment, with three technical replicates per biological replicate.

## 5. Conclusions

This study presents the first genome-wide identification of the bZIP transcription factor family in the woody plant *Juglans mandshurica*, characterizing 80 members classified into 13 subgroups. Evolutionary analysis indicated that three orthologous gene pairs between *J. mandshurica* and *Juglans regia* may have undergone positive selection following their divergence. Analysis of promoter cis-regulatory elements revealed an enrichment of stress-related elements, such as MYB and MYC, in *JmbZIP* genes. Expression profiling showed that subgroups S, A, and D were active across multiple physiological processes and light stress responses, indicating their potential involvement in these processes. Furthermore, qRT-PCR validation under salt and drought stress confirmed significant upregulation of key ABA signaling genes (*JmNCED3*, *JmABI5*, and *JmbZIP42*) and showed that their expression patterns were consistent with those of the functional downstream genes *JmRAB18* and *JmP5CS1*, suggesting their possible roles in stress responses. Notably, *JmbZIP41* and *JmbZIP42*, both from subgroup J, exhibited opposing expression responses to stress, suggesting possible divergence within this subgroup. The key *JmbZIP* genes identified in this study provide valuable genetic resources for stress resistance breeding in forest trees, with *JmABI5* and *JmbZIP42* representing promising candidates for enhancing stress tolerance.

## Data Availability

The raw genome sequence data and the RNA-seq datasets analyzed in this study are publicly available. The genome data can be accessed via the Genome Warehouse (NGDC) under accession number PRJCA006358 (https://ngdc.cncb.ac.cn/bioproject/browse/PRJCA006358, accessed on 13 April 2025). The RNA-seq datasets are deposited in the NCBI SRA database under accession numbers PRJNA816294, PRJNA848847, and PRJNA805360. All processed data generated in this study are provided in the Supplementary Materials. Further inquiries can be directed to the corresponding author.

## Supplementary Materials

The following supporting information can be downloaded at https://www.mdpi.com/article/10.3390/ijms27135770/s1.
