# Supplementary material for: Genome-Wide Survey and Expression Profiling of bZIP Transcription Factors in Juglans mandshurica Reveal Candidate Genes Involved in Floral Development, Light Stress, and Drought/Salt Tolerance
Source: Int J Mol Sci. 2026 Jun 26;27(13):5770. doi: 10.3390/ijms27135770 (PMC13362365; doi:10.3390/ijms27135770)
Supplement: Supplementary file 1 [file ijms-27-05770-s001.zip › Supplementary Figures.pdf]

Supplementary Figures

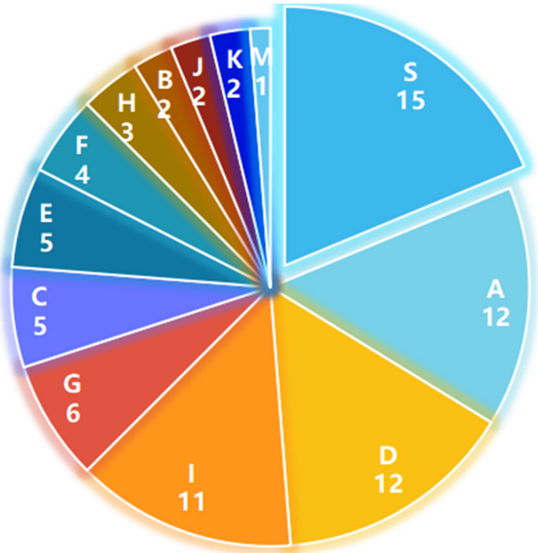

Figure S1. Gene count per phylogenetic subgroup of the bZIP gene family in *J. mandshurica.*

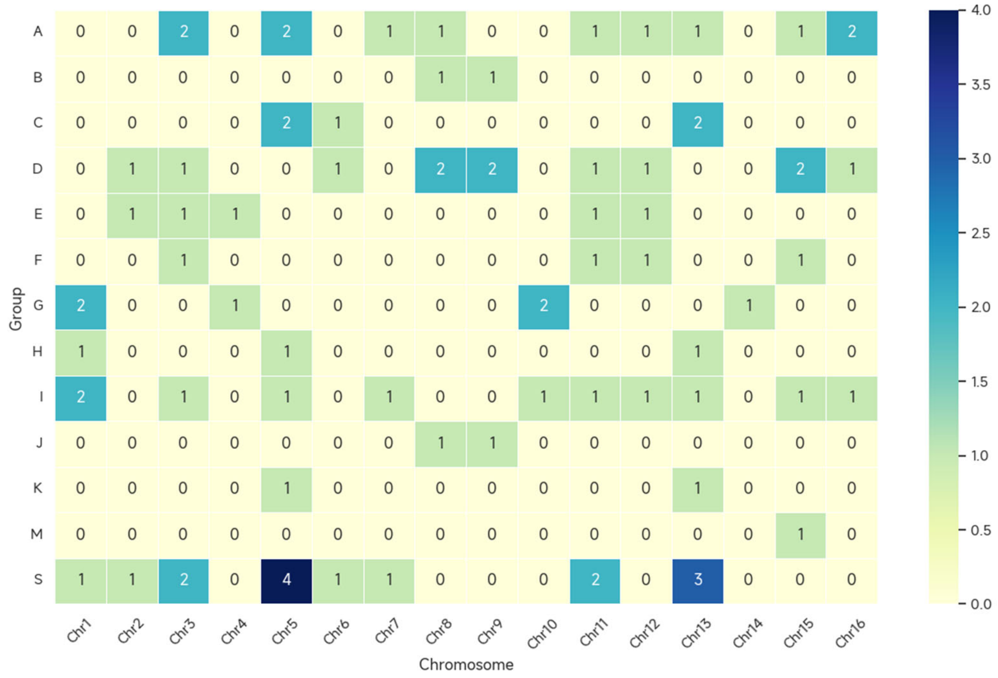

Figure S2. Chromosomal distribution of *JmbZIP* genes by phylogenetic subgroup in *J. mandshurica.*

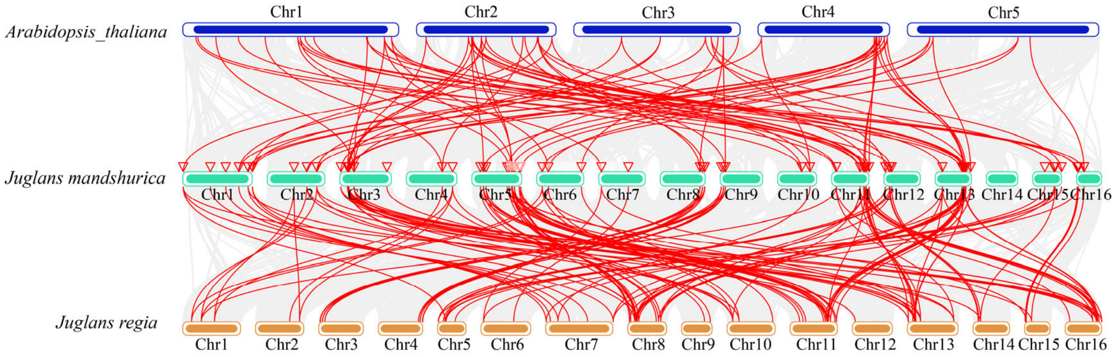

Figure S3. Synteny analysis of bZIP genes among *J. mandshurica*, *A. thaliana*, and *J. regia*. Gray lines indicate all systemic regions across the three genomes; red lines highlight systemic bZIP gene pairs.

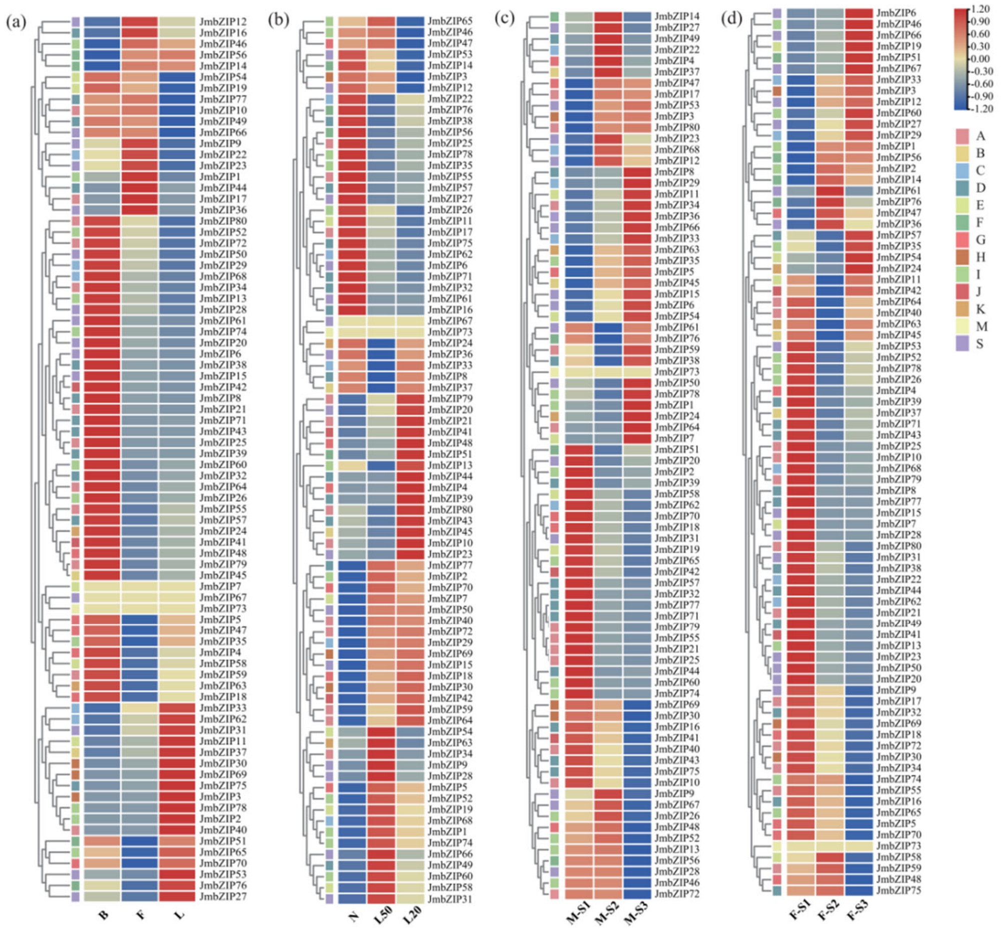

Figure S4. Expression profiles of the JmbZIP gene family across various conditions. (a) Expression in three tissue types: bark (B), green pericarp (F), and leaf (L). (b) Response to a 6-hour treatment at different light intensities (100%, 50%, and 20% of full sunlight). (c-d) Expression dynamics during three developmental stages of female (F-S1, F-S2, F-S3) and male (M-S1, M-S2, M-S3) flowers. The heatmap displays Z-score normalized expression levels for all identified *JmbZIP* gene family members. Phylogenetic subgroup affiliations are indicated by the color bar on the right.
